# Focal therapy will be the next step on prostate cancer management? | *Opinion:* Yes

**DOI:** 10.1590/S1677-5538.IBJU.2017.06.02

**Published:** 2017

**Authors:** Stênio de Cássio Zequi

**Affiliations:** 1 Editor Associado, International Braz J Urol; 2 Divisão de Urologia do A.C. Camargo Cancer Center Fundação A. Prudente, São Paulo, Brasil

**Keywords:** Therapeutics, Prostatic Neoplasms, Disease Management, Kidney Neoplasms

In the last decades, the main goal of the treatment of several solid malignancies was the maintenance of high cure rates, along with morbidity reduction. As occurred with the drastic reduction of radical mastectomies for breast cancer and popularization of nephron sparing surgeries for kidney cancer, winds for reducing the radicality of prostate cancer (PC) treatment are blowing the candles.

Despite of recent recommendations against PC screening, the high rates of overdiagnosis and overtreatment of PC patients are still observed and relevant. Nowadays, many of PC cases are diagnosed in early stages, comprising no more than 5 or 10% of the gland, and much of them are low or intermediate risk PC (1–3). However, for decades, the accepted treatments for all localized PC cases have not changed and were based in whole gland therapies (WGT), for example: radical prostatectomy, or radical external beam radiotherapy, brachytherapy, cryotherapy, HIFU or androgen deprivation (1–3).

Historically, all WGT result in excellent cancer control and high survival rates, however, these procedures are associated with high prevalence of urinary, sexual and intestinal side effects. These WGT's complications can negatively affect patients and their relatives' quality of life. In many cases, these side effects are severe, and additional high costs treatments are required.

Many PC new cases are indolent and/or clinically insignificant, and do not require active therapeutic intervention. For those patients, active surveillance (AS) and delayed intervention, proposed around 15 years ago, are well stablished as competitive, secure and ethical options, resulting in few urinary, sexual and intestinal side effects in short or mid-term follow-up (4). However, AS protocols can also generate inconveniences. They require patients adherence, multiple serum tests, medical visits, periodic MRI scans etc. that can result in psychological distress, and economic costs. Anxiety or depression due of a “non-treated cancer” can affect some individuals, or their partners or family. Additionally, against AS, there are the odds of tumor clinical/pathological understaging, and the risks of urinary tract infection, and lethal or fatal urosepsis associated with repeated prostate biopsies (1–4).

In summary, at present, for patients with low risk PC, it is absolutely ethical in one extreme to offer a WGT (with its side effects) or, in the other extreme, it is absolutely ethical to offer no intervention (AS). Philosophically, why not adopt a “middle term”, in which we could treat focally the cancer that affects the small percentage of the prostate (eliminating the cancer, as with WGT) and, at the same time, maintain under surveillance the rest of the gland (similarly to AS)? Could we with this approach reach high rates of cure as with WGT and preserve sexual, urinary and intestinal functions as with AS, without the concerns of an untreated cancer? These are the goals that are moving the researchers in favor to PC Focal Therapy (FT).

FT rationale has emerged with skepticism and critics, since PC is usually multifocal and heterogeneous, and only 17-40% of patients have unifocal tumor. On a first glance, FT can be administered only for this minority of patients. But the concept has expanded, and today the most accept rationale is to treat Index lesions (IL), and preserve the surrounding glandular tissue, that can be healthy, or with secondary lesions (SL) that can be submitted to surveillance, as in AS protocols.

Genetic sequencing studies of primary tumor and its respective metastases, performed after necropsies of patients who died due to advanced castration refractory PC, and several pathologic investigations, defined Index lesion (IL) as that with lethal clone of cells, that can originate the most aggressive metastases and are responsible for tumor aggressiveness and oncological outcomes (5, 6). Usually, IL hosts the highest histological grade of malignant cells, and is the largest lesion in the prostate (>0.5cc-1.3cc), being detectable by Multiparametric Magnetic Resonance Image (mpMRI). On the other hand, “secondary lesions” (SL) can co-exist in parallel with IL, but usually are microscopic or small lesions (undetectable at mpMRI), constituted by low grade tumors cells (Gleason ≤ 6), corresponding to “clinically insignificant tumors” and without aggressive cell clones, with minimal metastatic potential. In the majority of the cases, SL are not responsible for disease evolution/progression, being suitable for surveillance, without treatment (3, 5, 6).

FT was proposed almost 20 years ago, using Focal HIFU (high intensity focused ultra-sound), and Focal Cryotherapy. Initially, protocols were proposed for unilateral or single lesions, and included prostatic hemi-ablations; other authors proposed hemi-ablations plus contralateral apex or bases (“hockey stick fashion”), or even ablation of the whole gland, but sparing the neurovascular bundles, uni or bilaterally. With the progress of the devices, the side effects for adjacent organs have progressively reduced (as thermic damages in urethra or rectum that reduced dramatically with modern HIFU or Cryotherapy machines).

After treating only low grade tumors, the authors have expanded the indications for some intermediate risk PC (Gleason 7 tumor) and a few high grade (Gleason 8) tumors, but with favorable location. Since most patients with low risk tumors are suitable for AS, the more recent recommendations of experts (7) confirm that FT can be used in patients with localized intermediate risk PC (2). Patients with small focus of Gleason 7 or that with a large amount of Gleason 6 tumors are also fit for FT.

In a review in 2014 of 2350 men undergoing FT, 36% were classified as intermediate risk patients and 8% were high risk ones. Gleason 7 and 8 were found in 25% and in 4% of the patients, respectively (8).

A seminal question is the safety of margin around the tumor, that could configure a limitation of FT indication in apical or anterior lesions (particularly in lesions close to the capsule).

Beyond the scope of treatment of primary tumors, FT has been applied as salvage therapy for recurrent PC after external beam radiotherapy or brachytherapy. With the use of salvage FT, the side effects may be minimized in comparison with those of whole gland salvage methods. Wenske et al. have shown similar disease specific survival rates in 55 patients underwent salvage partial cryotherapy versus 273 underwent whole gland salvage cryotherapy after failure of radiotherapy or primary cryotherapy (9).

New FT strategies have emerged due to the best knowledge of natural history of PC, aside from the concepts of IL and SL, and modern multiparametric magnetic resonance image (mpMRI) and other image technologies (that generate tridimensional reconstructed imagens of the IL (in fusion with, for example, real time ultrasound)) and new devices, that can deliver energy in more specific and precise points of the gland. Based on these new technologies, it is possible to target only IL and around 25% of its surrounding tissue, in a security margin equivalent to 3-5mm, far from the “rims” of the IL. By the use of these procedures, other additional attractive possibilities include treatment of two IL lesions located in opposite regions of the prostate, with limited damage to non-tumor parenchyma.

According to a systematic review by Vallerio et al., in 2014, 49% of FT were performed with hemi-ablation or “hockey stick” ablation, 51% underwent focal ablation, and bilateral focal ablation was used in only 0.3% of patients. (8) There is an increasing tendency in favor of lesion ablation instead of hemi-ablation (7).

Reflecting the current approaches of the Scientific Community to FT, we can found in the web site *clinicaltrials.gov* (10) more than 40 trials investigating several aspects of FT, as in low volume localized disease, as a salvage therapy in United States. Similarly, researchers from United Kingdom and France are studying more than 10 trials on FT for PC. In the pubmed.gov web site, (11) when we insert the mesh term “prostate cancer focal therapy” we find 1952 articles, with 812 of those published in the last 5 years.

The more used surrogates to define biochemical control of FT are based on ASTRO or Phoenix criteria used for external beam radiotherapy and brachytherapy. In a recent review (2), the ranges of biochemical recurrence free survival (BRFS) for Focal Cryotherapy, Focal HIFU, Focal Lasertherapy and focal brachytherapy were respectively: 71-98%; 72-90%, 88% and 92% The presence of cancer in treated sites ranged from 5% to 30%, in the majority of these series, but only around 10- 15% of these cases had clinically significant tumors. Secondary treatments were used in around 25% of the patients (2).

In the largest cohort of Brachytherapy, with 1160 patients submitted to focal cryotherapy, the 3-years BRFS (ASTRO criteria) was 75% (12); urinary incontinence was detected in less than 4% of patients and sexual dysfunction in 42% (13).

In 2012, Ahmed et al. reported preliminary results of a prospective trial using focal HIFU in 20 patients (median PSA of 7.3ng/mL, and 75% of intermediate risk). After 3 months, 80% of the patients presented significant PSA reductions. Negative biopsy was verified in 18 of 19 patients, erectile function was preserved in 95% of the patients, and only 5% of the individuals were using pads for non-severe urinary incontinence. At 12 months of follow-up, 89% achieved the “trifecta”. In a larger population, the same authors reported no incidence of cancer in 30 of 39 patients biopsied after 6 months. And 92% of these patients were free of clinically significant PC. Four patients were re-treated. After 6 months, 100% of the patients were classified as “pad free” or “leak free” continence. Erections sufficient for penetration during sexual intercourse was present in 89%, intake of inhibitors of 5 phosphodiesterase drugs was reported in 45%. Trifecta was scored in 84% of these men (14).

Researchers, industry and their investors are concentrating efforts in developing new FT therapy devices, reinforcing the search for lesser aggressive PC treatment modalities.

At present, there are several FT equipment's as: Focal HIFU, Focal cryotherapy, laser ablation, interstitial laser thermotherapy, photodynamic therapy, irreversible electroporation, focal brachytherapy, focal radiotherapy, nanoparticles thermotherapy, interstitial thermal microwave therapy and interstitial radiofrequency ablation.

In relation to surgeons, new propositions of FT in prostate cancer surgery are also evolving. Recently, Villers et al. reported, for the first time, early results and complications rates of robotic partial prostatectomy, in the treatment of anterior apical tumors (15, 16) that are not suitable for the above mentioned FT modalities. Although we must have to wait for more data in this field, they confirm that FT will be one of the next steps for the treatment of primary low/intermediate risk, low volume, or recurrent localized PC.

Several concerns regarding FT have been questioned as: How to identify precisely the target area and its safe margins? How to deliver more precisely the energy? How to follow-up patients after therapy? What would be the best PSA level after treatment (since there are normal parenchyma that continues to produce this marker)? How to detect therapeutic failures? What would be the success and of salvage treatments applied for FT recurrences? What about the costs of FT?. It is necessary to differentiate recurrences from new lesions, and recurrences from new treatments etc.

As in almost all other aspects of prostate cancer, open questions still remain. Despite of these, new conservative treatment of this malignance is warranted and FT will be better understand with time. In this new era, for small low risk tumors, AS will continue to be the best approach; for locally advanced and high risk tumors, WGT (with or without multidisciplinary approach) will continue to be the preferred options. Between these two groups, there is a large amount of men with PC that will benefit from focal treatment of their tumors.
